# The Treatment of Hepatic Abscess

**Published:** 1902-12-13

**Authors:** 


### THE TREATMENT OF HEPATIC ABSCESS.

Lieutenant-Colonel W. K. Hatch, I.M.S., then read a paper on "Hepatic Abscess," a large number of cases of which he had seen in India. He especially drew attention to the fact that aspiration was attended with considerable danger, and ought only to be employed when the diagnosis had been established and it remained only to determine the precise locality of the abscess. Dangerous and even fatal hæmorrhage might be caused by such puncture, which in some cases might enter even the vena cava. His practice was to open the abdomen by a two-inch incision, exposing the liver, by which means one was enabled to introduce the canula with safety. He had no doubt that the proper treatment was incision, and the earlier the better, although it might be well in large abscesses to draw off part of the pus first, making the incision a day or two later. It was better, he said, to open the abscess by an incision between the ribs rather than below them, and he never irrigated the abscess cavity. The author of the paper did not seem to take much account of the pus escaping into the peritoneum in the course of the operation, and he did not specially suture the pleura to the thoracic wall to protect the pleural cavity. Several of the speakers, however, advocated careful packing of the wound to prevent infection by the escaping pus. But the main point of the paper was to insist upon the advantage of opening the abdomen and the danger of exploring with a needle in search of an abscess, especially in cases in which there might not be any abscess at all, for it was in these cases that such a proceeding might do most harm.

